# Supplementary material for: Catalytic Reduction of Dyes and Antibacterial Activity of AgNPs@Zn@Alginate Composite Aerogel Beads
Source: Polymers (Basel). 2022 Nov 9;14(22):4829. doi: 10.3390/polym14224829 (PMC9698220; doi:10.3390/polym14224829)
Supplement: Supplementary file 1 [file polymers-14-04829-s001.zip › polymers-1983112-supplementary.pdf]

Catalytic reduction of dyes and antibacterial activity of AgNPs@Zn@Alginate composite aerogel beads.

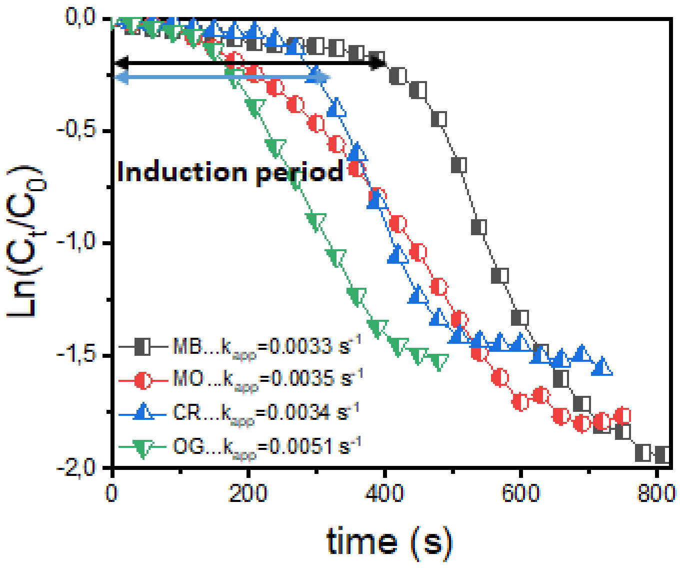

Figure S1. Plot of $Ln(C_t/C_0)$ versus time.

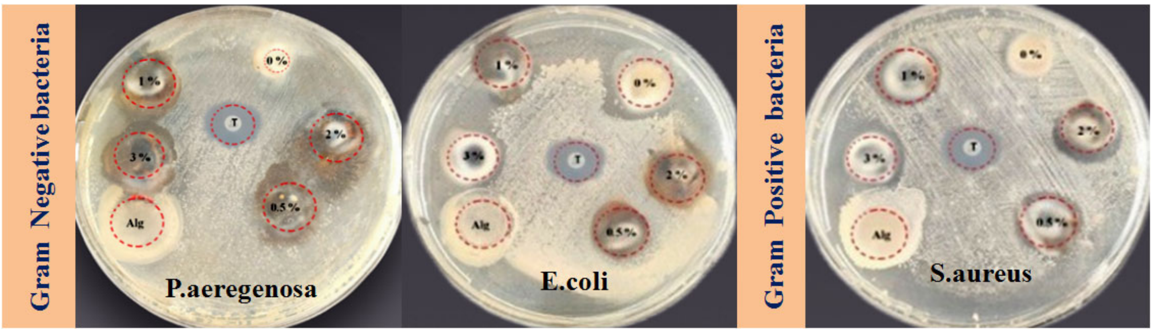

Figure S2. Digital images for antibacterial tests of different samples of Zn-ALG(AgNPs).

Table S1. Comparative study with materials carried out in the literature.

	catalyst	Catalyst mass (mg)	[dye] (mM)	[$NaBH_4$]	Reaction time (min)	k_{app} (min^{-1})	Ref
MB	Alg-St/AC1@CuNPs	30 mg	0.08	0.2 M	2.5 min	0.73	[31]
	Alg-St/AC5@CuNPs	30 mg	0.08	0.2 M	6.5 min	0.19	[31]
	Alg-St/AC10@CuNPs	30 mg	0.08	0.2M	5 min	0.3	[31]
	Ag^0@CH/PG	0.1g	0.07	0.2M	25 min	//	[34]
	**Zn-ALG(Ag 0.5%)**	5.3mg	0.1	8mM	7.5 min	0.198	**This study**
CR	Alg-St/AC1@CuNPs	30 mg	0.08	0.2 M	8 min	0.13	[31]
	Alg-St/AC5@CuNPs	30 mg	0.08	0.2 M	7 min	0.13	[31]
	Alg-St/AC10@CuNPs	30 mg	0.08	0.2M	4 min	0.36	[31]
	Fe^0-Cu^0/Alg-ACBDs	9.5 mg	0.07	0.2 M	6 min	0.655	[37]
	CuAg@BP-NaY	1 ×0.5 cm^2	0.05	0.1 M	4 min	0.64	[33]
	Ag@BP-NaY	1 ×0.5 cm^2	0.05	0.1 M	5 min	0.46	[33]
	Cu@BP-NaY	1 ×0.5 cm^2	0.05	0.1 M	7 min	0.31	[33]
	**Zn-ALG(Ag 0.5%)**	5.3mg	0.1	8mM	12.5min	0.204	**This study**
MO	Fe^0-Cu^0/Alg-ACBDs	9.5mg	0.07	0.2 M	4 min	1.163	[37]
	Ag^0@CH/PG	0.1g	0.07	0.2 M	10min	//	[34]
	CuAg@BP-NaY	1 ×0.5 cm^2	0.05	0.1 M	2 min	1.75	[33]
	Ag@BP-NaY	1 ×0.5 cm^2	0.05	0.1 M	4 min	0.78	[33]
	Cu@BP-NaY	1 ×0.5 cm^2	0.05	0.1 M	5 min	0.64	[33]
	**Zn-ALG(Ag 0.5%)**	5.3mg	0.1	8mM	12.5 min	0.21	**This study**
OG	MC@CA(1) hydrogel beads	4.8mg	0.6	0.6M	14min	//	[36]
	MC@CA(1) aerogel beads	4.8mg	0.6	0.6M	14 min	//	[36]
	**Zn-ALG(Ag 0.5%)**	5.3mg	0.1	8mM	8min	3.267	**This study**
